# Altered Onset Response Dynamics in Somatosensory Processing in Autism Spectrum Disorder

**DOI:** 10.3389/fnins.2016.00255

**Published:** 2016-06-08

**Authors:** Sheraz Khan, Javeria A. Hashmi, Fahimeh Mamashli, Hari M. Bharadwaj, Santosh Ganesan, Konstantinos P. Michmizos, Manfred G. Kitzbichler, Manuel Zetino, Keri-Lee A. Garel, Matti S. Hämäläinen, Tal Kenet

**Affiliations:** ^1^Department of Neurology, Massachusetts General HospitalBoston, MA, USA; ^2^Athinoula A. Martinos Center for Biomedical Imaging, MGH/MIT/HarvardBoston, MA, USA; ^3^Harvard Medical SchoolBoston, MA, USA; ^4^McGovern Institute for Brain Research, Massachusetts Institute of TechnologyCambridge, MA, USA; ^5^Department of Computer Science, Rutgers UniversityPiscataway, NJ, USA; ^6^Department of Radiology, Massachusetts General HospitalBoston, MA, USA; ^7^Department of Neuroscience and Biomedical Engineering, Aalto University School of ScienceEspoo, Finland

**Keywords:** autism spectrum disorders (ASD), magnetoencephalography (MEG), somatosensory cortex, feedforward, feedback, tactile sensing, cortical connectivity, biomarker

## Abstract

Abnormalities in cortical connectivity and evoked responses have been extensively documented in autism spectrum disorder (ASD). However, specific signatures of these cortical abnormalities remain elusive, with data pointing toward abnormal patterns of both increased and reduced response amplitudes and functional connectivity. We have previously proposed, using magnetoencephalography (MEG) data, that apparent inconsistencies in prior studies could be reconciled if functional connectivity in ASD was reduced in the feedback (top-down) direction, but increased in the feedforward (bottom-up) direction. Here, we continue this line of investigation by assessing abnormalities restricted to the onset, feedforward inputs driven, component of the response to vibrotactile stimuli in somatosensory cortex in ASD. Using a novel method that measures the spatio-temporal divergence of cortical activation, we found that relative to typically developing participants, the ASD group was characterized by an increase in the initial onset component of the cortical response, and a faster spread of local activity. Given the early time window, the results could be interpreted as increased thalamocortical feedforward connectivity in ASD, and offer a plausible mechanism for the previously observed increased response variability in ASD, as well as for the commonly observed behaviorally measured tactile processing abnormalities associated with the disorder.

## Introduction

Autism spectrum disorder (ASD) is diagnosed by hallmark abnormalities in social behavior, and has a complex genetic basis (Berg and Geschwind, 2012; Skafidas et al., 2014; Pramparo et al., 2015) with no clear disease etiology. The neural correlates of ASD have been extensively explored, using a wide range of paradigms and non-invasive neuroimaging methods. One of the more consistent findings in ASD is that the connectivity between different brain areas is abnormal in ASD (Khan et al., 2013). This has been explored using both anatomical connectivity measures (Wolff et al., 2012; Mueller et al., 2013; Peeva et al., 2013) and functional connectivity measures (Kana et al., 2011; Müller et al., 2011; Wass, 2011; Vissers et al., 2012).

The prevailing hypothesis in the field (Rubenstein and Merzenich, 2003; Just et al., 2004), has been that long-range functional connectivity is reduced and local functional connectivity is increased in ASD (Belmonte et al., 2004; Minshew and Williams, 2007). However, evidence for this dual hypothesis is inconclusive. In particular, the hypothesis that long-range functional connectivity, i.e., connectivity between two spatially distinct brain regions, is universally reduced in ASD has been challenged by recent studies showing instances of both increased (Cerliani et al., 2015) and normal (Tyszka et al., 2014) long-range functional connectivity in ASD.

Previously, we proposed that the inconsistencies in long-range functional connectivity studies in ASD might be reconciled if the directionally of the connectivity, i.e., the direction in which two areas are connected, would be considered. Specifically, we proposed that long-range feedforward (bottom-up along the cortical hierarchy) connectivity would be abnormally increased in ASD, while feedback (top-down along the cortical hierarchy) long-range connectivity would be abnormally reduced (Khan et al., 2015; Kitzbichler et al., 2015). In particular, in our recent study of cortical responses to vibrotactile stimuli in ASD, we showed that long-range functional connectivity was indeed significantly increased in the ASD group in the feedforward direction, from the primary somatosensory cortex (S1), upwards toward the secondary somatosensory cortex (S2) (Khan et al., 2015).

In that same study, we also found a significantly increased onset response in S1 in the ASD group. While the response in S1 was significantly increased at onset in the ASD group, it was not possible to determine, based on our prior analysis, whether this increase was generated locally, or via abnormal long-range connectivity, such as reduced feedforward functional connectivity from the thalamus for instance. This question is important, because increased local connectivity and increased long-range functional connectivity might have a similar final signature in the cortex, but would be generated and mediated by substantially different neural mechanisms, and thus different neural abnormalities. Thus, delineating the neural mechanisms that underlie the observed abnormal response in ASD is absolutely essential for understanding the abnormal neurophysiology of ASD.

To address this question, we focused here on the transient component of the response, and specifically on the rising edge of the evoked response. This transient response window, 30–70 ms immediately following the onset of the cortical response, has not been previously studied in relation to abnormal tactile processing in ASD. Given its timing, this part of the response is most likely generated at least in part by feedforward inputs from the thalamus. However, the mere observation of an increased response amplitude during that period is not sufficient to indicate whether the processes leading to that increase are local, or generated by long-range connections. Here, to test our hypothesis, that the increase in the transient evoked response observed in ASD is due to feedforward inputs from subcortical regions, we applied a novel measure that indicates how activation of a small neural population spreads in adjoining areas to become locally synchronized (Khan et al., 2009). This method, which is referred to as Spatio-Temporal Divergence (S-T Div), uses techniques based on the concept of optical flow, and was recently adapted to map the time-course of spatiotemporal propagation of brain activity across different cortical region (Khan et al., 2011).

## Results

### Spatial localization of evoked response to tactile vibrations

**Figure 1:**
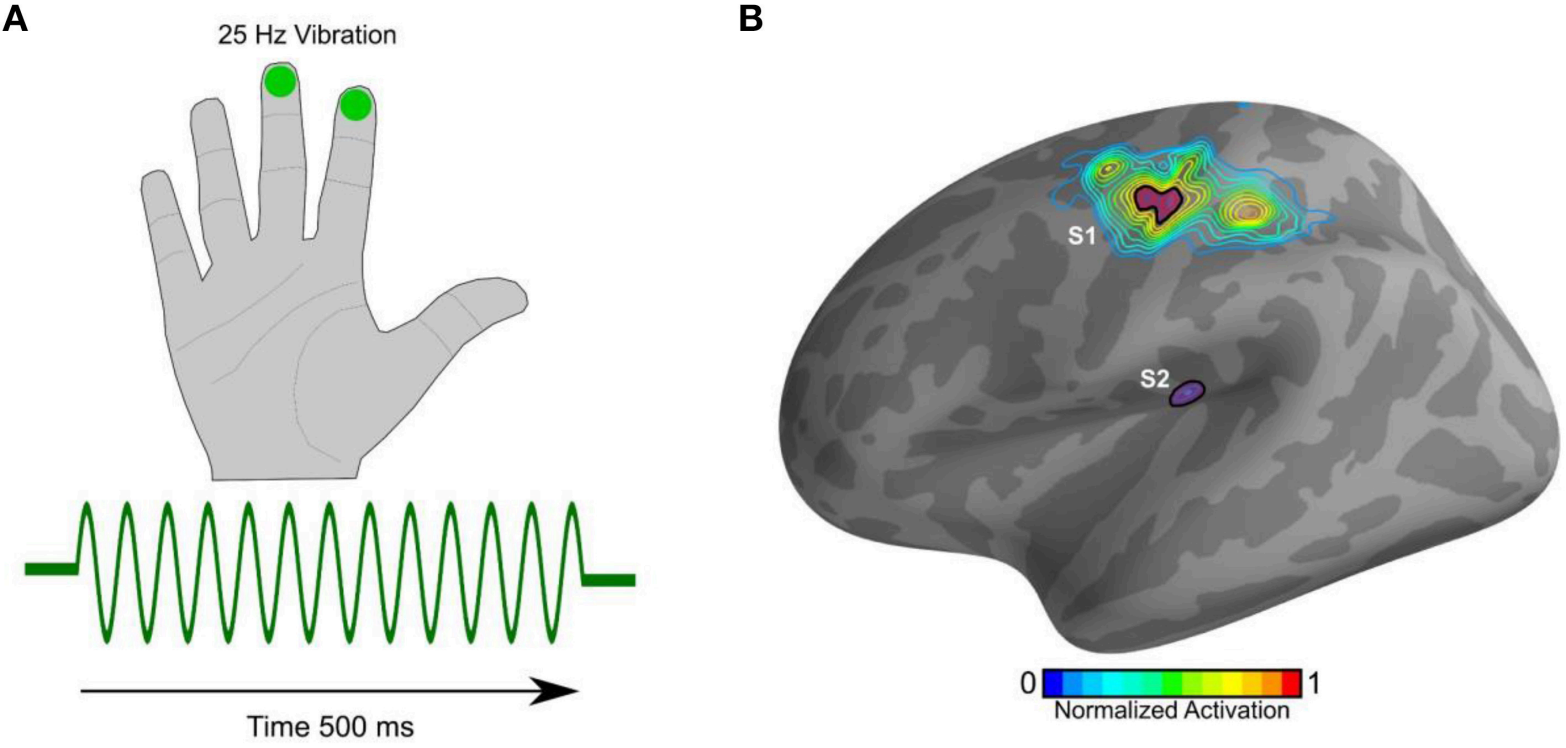
**Stimulus and source localization**. **(A)** 500 ms train of pulses at 25 Hz (green trace) was delivered via a pneumatic stimulator and experienced as gentle vibrations on the index and middle right fingers. **(B)** The estimated cortical sources showing activation in S1 and S2. The contour plot represents average activation on the cortical manifold. The distance between adjacent contours is 10%.

As expected and as described previously (Khan et al., 2015), the cortical evoked responses to the 25 Hz vibrotactile stimulus (Figure 1A) localized to the contralateral (left) S1 and S2 (Figure 1B).

### Sharper evoked response in ASD

**Figure 2:**
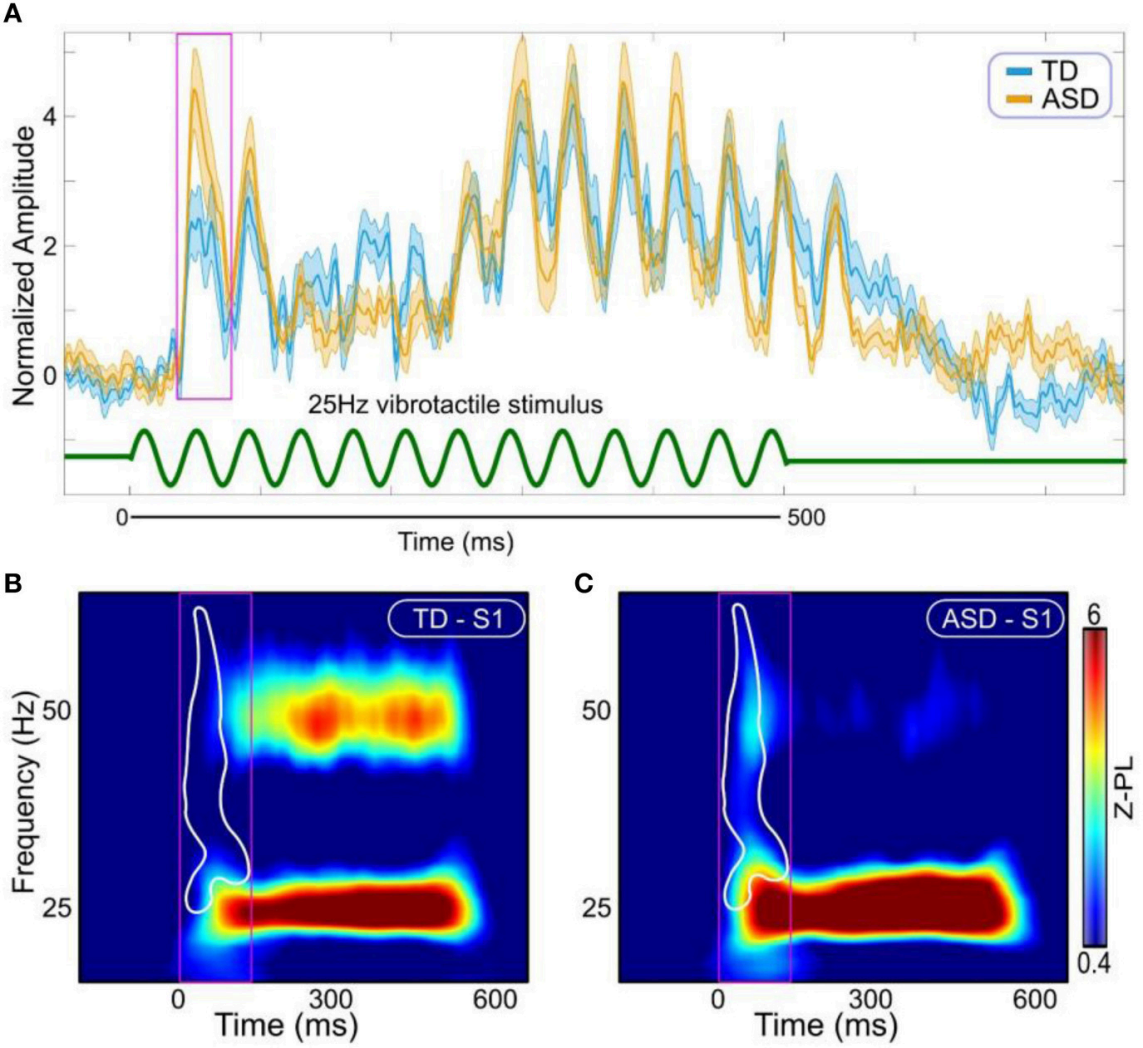
**Evoked responses. (A)** Evoked responses in S1 (Orange ASD; Cyan TD). Stimulus is represented with green curve at the bottom. Magenta box shows the window for the first transient peak [30–70 ms]. **(B)** Time-frequency representations of Z-scored phase locking (Z-PL) at S1 in the TD group. **(C)** Time-frequency representations of Z-scored phase locking (Z-PL) at S1 in the ASD group. White contour outlines the region where the response was significantly increased in the ASD group (*p* = 0.0470, cluster corrected). Magenta boxes show time window for the transient response in the time-frequency domain [0–140 ms].

There was no group difference in the latency of the response. The amplitude of the evoked transient response was slightly increased in the ASD group relative to the TD group, but this difference was not statistically significant (Figure 2A). In contrast, when the cortical response was examined over the onset time window in the time-frequency domain, i.e., with spectral specificity rather than averaging over the frequency domain as for the standard evoked response shown in Figure 2A, significant group differences emerged (Figure 2B, *p* = 0.0470, corrected). The difference arose primarily from the higher frequencies, at the 25–60 Hz range.

### Increased onset response divergence in ASD

**Figure 3:**
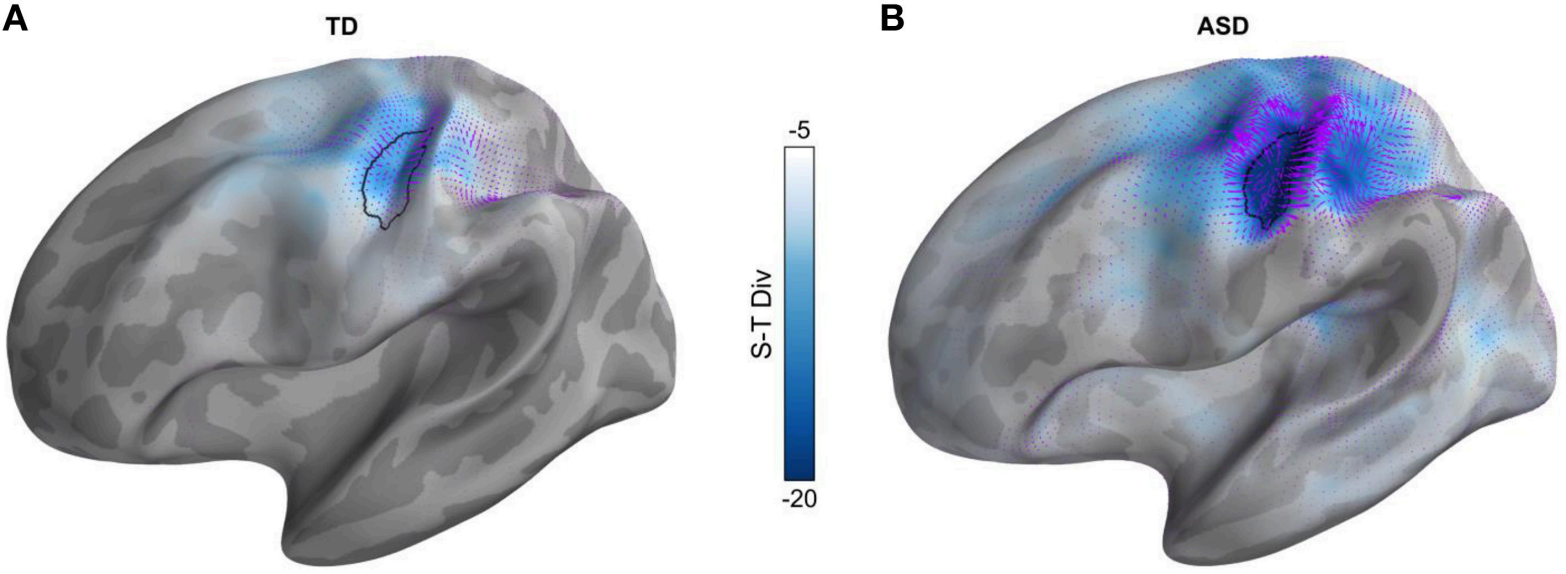
**S-T Div during the onset of the transient tactile response**. S-T DIV in **(A)** The TD group, **(B)** The ASD group. The colormap represents the magnitude of divergence, and the purple vectors represent the velocity of the divergence. Black outline represents the area that is statistically significantly different (*p* = 0.034, cluster corrected) between the TD and ASD groups.

We computed the spatio-temporal divergence (S-T Div) at the onset component of the response, and specifically at the rising edge of the first peak (30–70 ms). This was done by selecting the latency for each subject individually, computing S-T Div for that particular subject at their latency, and then averaging the results at the group level. At this time window, the ASD groups demonstrated significantly increased S-T Div in S1 (Figure 3, *p* = 0.034, corrected). As a control, we also examined S-T Div during the steady state component of the response (*t* = 250–550 ms). As expected, there were no significant group differences in this later time window.

### Correlations with behavioral measures and prior neurophysiological measures

**Figure 4:**
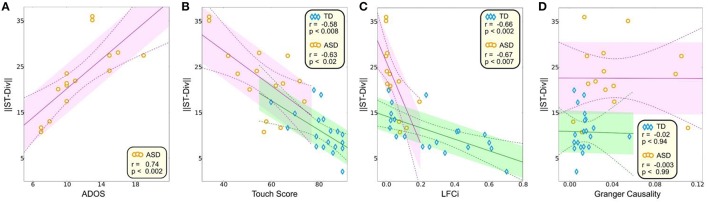
**Correlations between S-T Div and other measures**. Correlation between S-T Div and: **(A)** ADOS score, **(B)** Touch score, **(C)** LFCi, and **(D)** GCS. The shaded areas (TD in green, ASD in purple) delineate the standard error, and the dashed lines encompass 95% of the confidence interval for the correlation.

The neurophysiologically derived S-T Div was negatively correlated with the behaviorally derived ADOS (ASD group, *P* < 0.002, *r* = 0.74, Figure 4A) and touch perception score (TD group, *P* < 0.008, *r* = −0.58; ASD group, *P* < 0.02, *r* = −0.63, Figure 4B). Because the participants are identical to those in our prior study (Khan et al., 2015), we also assessed whether the onset derived S-T Div correlated with the steady-state derived neurophysiological measures from our prior study. Our steady state measures consisted of the LFCi (“Local Functional Connectivity index”), which estimated local functional connectivity in S1 during the steady state component of the response, and the GCS (Granger Causality score), which estimated the strength of feedforward connectivity from S1 to S2 during the steady state component of the response. Both were abnormal in the ASD group, with LFCi abnormally decreased, and the GCS abnormally increased. Our correlation analysis showed that LFCi was correlated with S-T Div for both the TD (Figure 4C, *P* < 0.002, *r* = −0.66) and ASD (*P* < 0.007, *r* = −0.67) groups. In contrast, S-T Div was not correlated with the GCS (Figure 4D).

### Statistical classification

**Figure 5:**
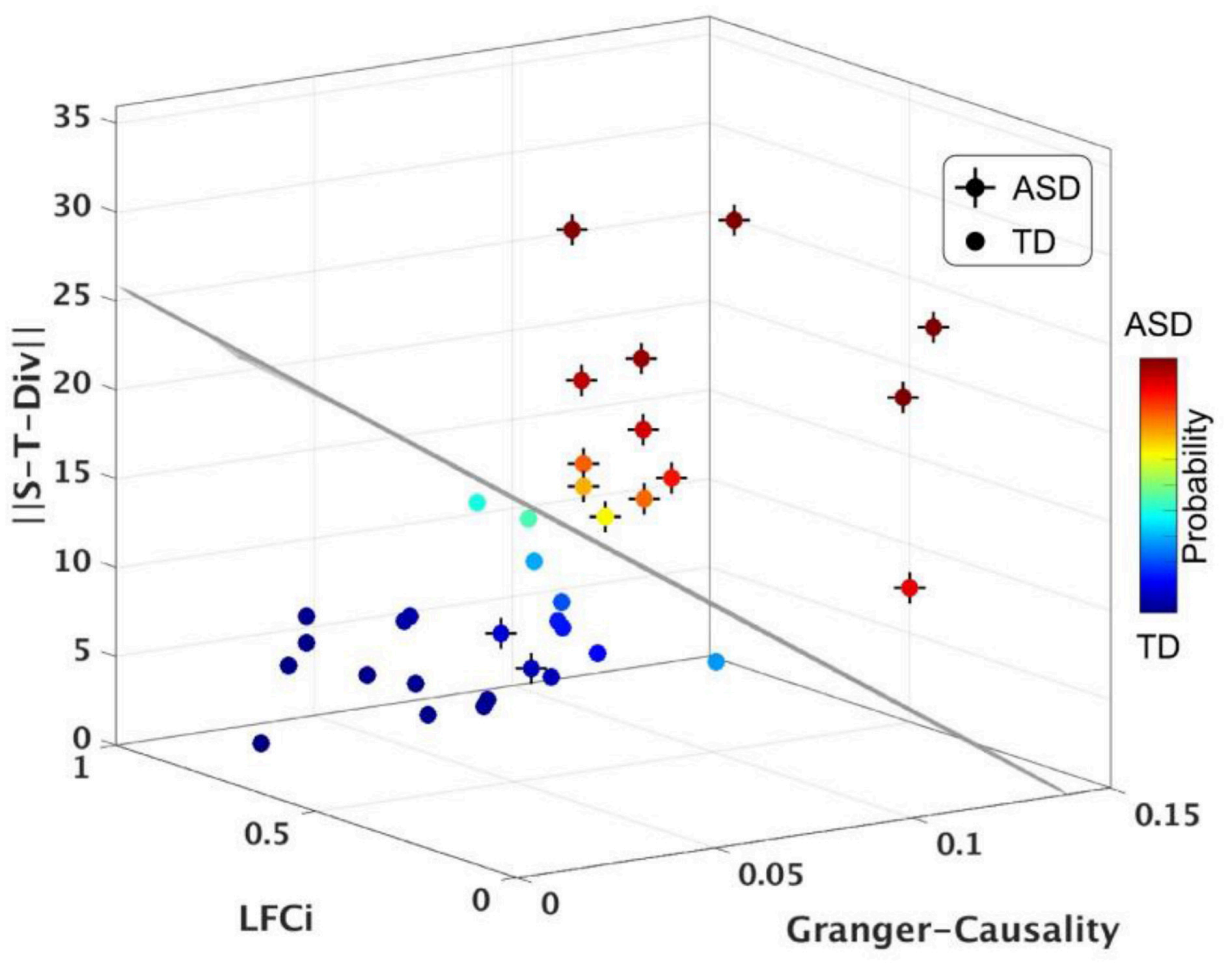
**Classifier results, using S-T Div, LFCi and Granger Causality**. Visualization of LDA analysis using the full dataset. Each axis corresponds to each neurophysiological imaging feature. The probability of a participant having a diagnosis of ASD is shown as color of the sphere. Plain sphere represents the TD participants, while sphere with a cross represent ASD participants. The black line represents classification boundary (see also, Figure S1 and Movie M1).

Lastly, we tested whether S-T Div could be used to blindly classify participants with ASD (neuroimaging Biomarker) from TD participants, using a Linear Discriminant Analysis classifier (LDA). This approach evaluates the sensitivity and specificity, and thus the relevance, of the assessed neurophysiological measure to the behavioral phenotype. Using S-T Div alone, the classifier had 83.3% accuracy (80% sensitivity, 90% specificity). We then repeated the classifier computations using S-T Div alongside our two previously derived neurophysiological measures, LFCi, and GCS. The combination of these three neurophysiological features yielded a mean classification accuracy of 91.6%, with 95% specificity and 90% sensitivity (Figure 5, Figure S1 and Movie M1). In our prior work the accuracy of the classifier was 89.7%. To assess whether adding the S-T Div measure significantly improved the classifier, the prior model (using LFCi, GCA) and the current model (using LFCi, GCA, S-T Div) were compared using the Akaike Information Criterion (AIC). The AIC score was −79.98 for the first model, and −92.17 for the second model. These scores, with a greater than 12-point difference, indicate that adding S-T Div significantly improved the model.

## Discussion

In the vast majority of studies, abnormal functional connectivity in ASD and abnormal evoked responses in ASD have been addressed separately. It is clear that functional connectivity and evoked responses are not independent from one another, but instead are tightly coupled. In our prior study using the same paradigm (Khan et al., 2015), we showed that the observed increases in steady state responses in the ASD group at 25 Hz in S2, were due to increased feedforward connectivity from S1. We also hypothesized that the observed increased onset response in S1 was due to increased feedforward connectivity from the thalamus, but were not able to test this hypothesis at the time.

The current method (S-T Div) allowed us to test this hypothesis indirectly, since it measures the flow (magnitude and velocity of spread) of neural activation in a given region and time window. The velocity at the onset of the response in S1, at the rising edge of the response, before local connections are strongly activated through recurrent loops, is likely to arise entirely or nearly entirely from feedforward connections into S1, primarily from the thalamus. While an increase in magnitude might arise from local recurrent connections, an increase in the velocity of spread can be attributed with relatively high certainty to an increase in feedforward inputs (Papadelis et al., 2012). Indeed, we found that at rising edge of the transient response in S1, this flow was greatly and significantly increased in ASD relative to TD.

Interestingly, as is evident from the time-frequency plots presented in Figure 2B, the evoked response in S1 in ASD is abnormally increased not only at the 25 Hz component of the response, but also at higher frequencies, including the 50 Hz component of the response. This seemingly contradicts our prior results. In our prior study (Khan et al., 2015), using a computational model and prior literature, we argued that only the 25 Hz component of the response, which was increased in ASD, is generated via feedforward connectivity, while the steady state of the 50 Hz component of the response, which was reduced in ASD, is generated via local connectivity within S1 and its immediate vicinity, i.e., horizontal connections across layers II/III. Simply put, why would the response in higher frequencies, and specifically around 50 Hz, be increased in ASD in the transient component immediately following the onset, but decreased during the steady state component of the response? If both the transient onset component and the steady state component of the cortical response were generated by the same neural mechanisms (local recurrent connections), the interpretation of the 50 Hz component of the response we proposed earlier would be inconsistent with the current proposed interpretation.

The logical resolution of this apparent conflict emerges from a line of studies affirming the fundamentally different nature of the onset component of the response relative to the steady state component (Nangini et al., 2006). For instance, somatosensory inputs from the thalamus to area 3b have been shown to evoke fast and slow adapting response patterns in non-human primates where one set of cortical cells respond only to stimulus onset and offset, while the other module respond throughout stimulus presentation (Sur et al., 1984). In contrast, the steady state response serves to more linearly convey detailed information about attended stimulus features (Ramcharan et al., 2005; Sherman, 2012). Furthermore, the corticothalamic pathways that would be most active during the onset component of the response, are largely distinct from the interareal corticocortical pathways that would be most active during the steady state component of the response (Petrof et al., 2012). Thus, the opposite patterns we observed in ASD for the onset component and the steady state components of the response around 50 Hz are not contradictory, as they are probably generated by at least partially independent neuronal assemblies.

That said, it is worthwhile to note that the strong correlation we observed between S-T Div and LFCi suggests that while these two temporally differentiated components of the response are distinct, they are not independent. However, from the current data, it is not possible to determine to what extent the abnormal response in ASD during the steady state component of the response is influenced by the initial abnormality in the onset component of the response. Since the two measures, S-T Div and LFCi, are correlated but not perfectly so, it is plausible that the reduced steady state response in ASD is a result both of the state of the neuronal assemblies following the increased onset response, alongside the previously discussed (Khan et al., 2015) inherent abnormalities in the local networks that mediate the steady state component of the response. Furthermore, the results from our classifier analysis indicate that the S-T Div analysis of the onset period adds independent information to the prior analyses of the steady state component of the response.

The differentiation proposed here between the feedforward dependent onset component of the response and the local feedback dependent steady state component of the response, is in line with studies of ASD that indirectly infer increased bottom-up perceptual processing tendencies in ASD (Neumann et al., 2006; Jarvinen-Pasley et al., 2008; Cook et al., 2012; Amso et al., 2014; Robertson et al., 2014). They are also in line with prior fMRI-based studies finding increased thalamocortical connectivity in ASD, in paradigms that were more likely to activate feedforward networks (Mizuno et al., 2006; Cerliani et al., 2015). These results are also intriguing in the context of a recent finding of increased inter-trial variability in ASD (Dinstein et al., 2012). Unmodulated, i.e., inconsistently gain controlled, feedforward inputs, as observed previously in ASD (Peiker et al., 2015), would likely result in more variable trial to trial onset responses. Lastly, these results are also relevant in the context of the high prevalence of behavioral sensory hypo- and hyper- sensitivities in ASD (Tommerdahl et al., 2007; Marco et al., 2011, 2012). Increased feedforward inputs and flow of sensory information would naturally result in hyper-sensitive behavior. It is possible that the observed hypo-sensitivities are due to generalized down regulation, as a compensatory strategy to the increased input intensities. Such a compensatory strategy would likely result in hypo-sensitivities.

An important limitation of the study is that this method does not directly measure thalamocortical feedforward connectivity from the specific thalamic nuclei, since no thalamic activation has been observed directly. Thus, the proposed interpretation, while relying strongly on known properties of response onset in early sensory cortex, and while fitting well with other studies, remains an indirect interpretation. Alternatively, other processes may also impact the observed abnormal dynamics of the onset response. For instance, it has been suggested that excitatory feedforward drive and feedback input from higher-order cortex or non-specific thalamic nuclei might also contribute to the onset component of the response (Cauller and Kulics, 1991; Jones et al., 2009). In addition, local interactions between excitatory and inhibitory circuits that occur before the M70 peak (Peterson et al., 1995) may also impact the abnormal dynamics observed here.

In summary, in our previous studies (Khan et al., 2015; Kitzbichler et al., 2015), we found increased forward cortical functional connectivity in ASD during the steady state component of the cortical response, from S1 to S2. We also found an increased onset response in S1 in the ASD group. In the present investigation we used the novel S-T Div measure to assess the dynamics of the onset response in S1. The observed dynamics are consistent with an interpretation of increased feedforward thalamocortical connectivity. The interpretation proposed here of the result of the S-T Div measure, is consistent with the conjecture that stronger feedforward connectivity is likely characteristic of ASD, andmay underlie the behaviorally observed aberrant somatosensory and vibrotactile processing in ASD.

## Materials and methods

### Participants

Participants were 15 males diagnosed with ASD and 20 age-matched TD males, ages 8–18 (11.6 mean age). ASD participants had a prior clinically verified ASD diagnosis, met a cutoff of > 15 on the SCQ, Lifetime Version, and were assessed with either Module 3 (*n =* 3) or 4 (*n* = 12) of the ADOS (ADOS, Lord et al., 1999), administered by trained research personnel who had established inter-rater reliability. Individuals with autism-related medical conditions, e.g., Fragile-X syndrome, tuberous sclerosis, and other known risk factors, e.g., premature birth, were excluded from the study. All TD participants were below threshold on the SCQ and were confirmed to be free of any comorbid neurological or psychiatric conditions, and of substance use for the past 6 months, via parent and self-reports. The ASD and TD groups did not differ in verbal or nonverbal IQ, as measured with the Kaufman Brief Intelligence Test—II (Kaufman and Kaufman, 2004). Handedness information was collected using the Dean Questionnaire (Piro, 1998). Only right-handed participants were included in the study. Additional details on the participants are provided in Table T1. Participants overlapped in full with those studied in our prior publication on this paradigm (Khan et al., 2015). All the experimental protocols were approved by The Massachusetts General Hospital (MEG) Institutional Review Board and all procedures were carried out in accordance with the approved guidelines. Written informed consent was obtained from all subjects.

### Experimental paradigms and MEG data acquisition

Vibrotactile stimulation in the MEG consisted of pulses applied to the index and middle right fingers at 25 Hz using a custom made pneumatic tactile stimulator with latex tactor tips, based on a published design (Briggs et al., 2004). The duration of each stimulus train was 500 ms with an inter-stimulus interval of 3 s with a 500 ms jitter. The stimuli were presented while participants were watching a movie. Participants were instructed to not pay attention to the stimulation and not move their hands. Hands were kept still using an armrest, and a blanket positioned over the arm. The sequence of stimuli was presented using the psychophysics toolbox (www.psychtoolbox.org). A total of 100 trials were collected. The total recording time was 6 min per subject.

MEG data were acquired inside a magnetically shielded room (IMEDCO, Hagendorf, Switzerland) (Khan and Cohen, 2013) using a whole-head VectorView MEG system (Elekta-Neuromag, Helsinki, Finland), comprised of 306 sensors arranged in 102 triplets of two orthogonal planar gradiometers and one magnetometer. The MEG signals were acquired at 600 Hz, with a hardware bandpass filter set between 0.1 and 200 Hz. The position and orientation of the head with respect to the MEG sensor array was recorded continuously with help of four Head Position Indicator coils (Uutela et al., 2001; Zaidel et al., 2009). To allow co-registration of the MEG and MRI data, the locations of three fiduciary points (nasion and auricular points) that define a head-based coordinate system, a set of points from the head surface, and the sites of the four HPI coils were digitized using a Fastrak digitizer (Polhemus, Colchester, VT, USA) integrated with the Vectorview system. The ECG and EOG signals were recorded simultaneously to identify epochs containing heartbeats as well as vertical and horizontal eye-movement and blink artifacts. During data acquisition, on-line averages were computed from artifact-free trials to monitor data quality in real time. All off-line analysis was based on the saved raw data. In addition, 5 min of data were recorded from the room void of a subject before each experimental session for noise estimation purposes.

### Structural MRI data acquisition and processing

T1-weighted high-resolution magnetization-prepared rapid gradient echo (MPRAGE) structural images were acquired using a 3.0 T Siemens Trio whole body MR scanner (Siemens Medical Systems, Erlangen, Germany) and a 32 channel head coil. The in-plane resolution was 1 × 1 mm^2^, slice thickness 1.3 mm with no gaps, and a TR/TI/TE/Flip Angle 2530 ms/1100 ms/3.39 ms/7°. Cortical reconstructions and parcellations for each subject were generated using FreeSurfer (Dale et al., 1999; Fischl et al., 1999a). After correcting for topological defects, cortical surfaces were triangulated with dense meshes with ~130,000 vertices in each hemisphere. For visualization, the surfaces were inflated, thereby exposing the sulci (Dale et al., 1999).

### MEG data pre-processing

#### Cleaning and motion correction

The data were spatially filtered using the SSS method (Elekta-Neuromag Maxfilter software) to suppress noise generated by sources outside the brain (Taulu et al., 2004; Taulu and Simola, 2006). SSS also corrects for head motion between and within runs (Taulu et al., 2004). Cardiac and ocular artifacts were removed by signal space projection (Gramfort et al., 2013). The MEG data were then further low-pass filtered at 145 Hz to remove the HPI coil signals. The filtered data were then used for all further analyses.

#### Epoching

The data were epoched into single trials lasting 2.5 s, from 1000 ms prior to stimulus onset to 1500 ms following it. Epochs were rejected if the peak-to-peak amplitude during the epoch exceeded 1000 fT and 3000 fT/cm in any of the magnetometer and gradiometer channels, respectively. This resulted in the loss of 2–20 trials per participant. To maintain a constant signal to noise ratio across conditions and participants, the number of trials per condition per participant was fixed at 80, the minimum number of accepted trials that we had for each condition and participant. For participants that had more than 80 good trials, we selected 80 trials randomly from the available trials.

#### Transient response time window selection

For the standard evoked response (Figure 2A), we selected the first transient peak in the time window between 30 and 70 ms from stimulus onset, to evaluate latency and amplitude. For the response in the time-frequency domain, we needed to account for smoothing due to the convolution of the seven cycles complex Morlet wavelet with the data. Therefore, the time window of interest was 0–140 ms from stimulus onset.

### Data quality

There were no group differences in overall quality of the data, and the number of good (un-rejected) trials per condition was similar between groups and across conditions. For each participant, the same set of trials was used for all analyses.

### Mapping MEG data onto cortical space

#### Source estimation

The cortical source space consisted of 10,242 dipoles per hemisphere, corresponding to a spacing of approximately 3 mm between adjacent source locations. The forward solution was computed using a single-compartment boundary-element model (Hämäläinen and Sarvas, 1989). The individual inner skull surface triangulations for this model were generated with the watershed algorithm in FreeSurfer. The current distribution was estimated using the minimum-norm estimate by fixing the source orientation to be perpendicular to the cortex (Gramfort et al., 2014). The noise covariance matrix was estimated from data acquired in the absence of a subject prior to each session. We employed depth weighting to reduce the bias of the minimum norm estimates toward superficial currents (Lin et al., 2006).

#### Inter-subject cortical surface registration for group analysis

A morphing map to optimally align the cortical surface of each participant to an average cortical representation (FsAverage in FreeSurfer) was computed in FreeSurfer (Fischl et al., 1999b).

### Data analysis

#### Phase locking

Inter Trial Phase Locking (PL) is a method to quantify phase synchrony across multiple trials. To compute PL, we convolved the epoched time series with a dictionary of complex Morlet wavelets (each spanning seven cycles). We then normalized the resulting complex coefficients by dividing by their absolute magnitude and averaging the unit-norm phasors across trials for each time-frequency bin. We then took their absolute value so that each number ranged between 0 and 1, with 0 representing a uniform distribution of phase angles and 1 representing perfectly synchronized phase angles, across trials (Tallon-Baudry et al., 1996; Makeig et al., 2002). Mathematically PL is defined as:

$$PL(f,t)=\frac{1}{N}\left|\sum_{n=1}^{N}e^{\phi^{k(f,t)}}\right|$$

Where Ø^K^ represent instantaneous phase resulting from convolution of the trial with the complex Morlet wavelet, and N is the numbers of trials.

#### Z-PL (normalized phase locking)

To compute Z-PL (Figure 2), we compared each PL value to a set of surrogate null distributions, to correct for statistical biases proportional to the number of epochs. This approach is non-parametric, and makes no a-priori assumptions besides the independence across the trials in the experimental data. The independence across trials was motivated by the fact that there was an average 3 s time interval between trials, and anticipation effects were eliminated because our experimental paradigm had a 500 ms jitter in Stimulus-Onset Asynchrony. Z-PL was computed as follows: each trial was first circularly shifted by a random lag (τϵ(0, *T*], where T = period (1/f) in samples) and PL was computed on the shifted epoched data. This process was repeated 500 times. Z-PL was then computed by subtracting the mean and dividing by the standard deviation of the null distributions from the actual PL values.

### S-T div decomposition

S-T Div is composed of two components. The first is the scalar component of the extent of divergence of the source estimates, i.e. the magnitude of the divergence, illustrated in Figure 3 as a colormap. The second is the velocity of this divergence, illustrated in Figure 3 with purple vectors, to represent both direction and magnitude. The S-T Div decomposition involves two steps: (i) The optical flow of distributed MEG/EEG MNE normalized estimates, where the relative maximum is set to be one unit for each individual subject, is computed on the cortical manifold. This step ensures that amplitude does not impact the result, so that different data sets where signal to noise may not be constant, can nonetheless be directly compared. (ii) Helmholtz-Hodge decomposition is then applied to the optical flow computed previously. The details and mathematics of the approach were published previously (Khan et al., 2011). Briefly, optical flow V is a vector field which defines the motion of scalar quantity *I*, defined on a surface *M* and at time *t*, such that:

$$\partial_{t}+g\left(V,\nabla_{M}I\right)=0$$

Where *g*(.,.) is the scalar product, modified by the local curvature of *M*. Given optical flow vector field **V** defined on a surface *M*, there exists: a scalar field U, a rotational vector field A, and a harmonic vector field **H** such that:

V = ∇_M_U + ∇ x A + H The scalar field U is the divergnce of the scalar field *I*, and **V**_*div*_ = ∇_M_U is the divergence vector field component of vector field **V** defined on a surface *M* and at time *t*. Optical flow and S-T Div are availaible as part of open-source MEG/EEG toolboxes; Brainstorm (Tadel et al., 2011) and MNE-Python (Gramfort et al., 2013).

Lastly, it is important to note that S-T Div is not affected by the point spread of MNE solution. This is because S-T div is computed by taking the gradient in space and time. The point spread of MNE results from the regularization of the ill-posed inverse solution. Therefore, for a particular location in space, the spread is “constant” across different time points. Thus, because it is constant, taking the gradient cancels the impact of the point spread. This is discussed at length in prior publications on the topic (Khan et al., 2011).

### Correlations analyses

All correlation coefficients and the corresponding *P*-values were computed using Pearson correlation (Figure 4). Correlations resulting in significant *P*-values were then tested using Robust Correlation (Pernet et al., 2012), which strictly checks for false positive correlations using bootstrap resampling and 6 correlation tests (bootstrap Pearson correlation, bootstrap Spearman correlation, bootstrap Bend correlation, bootstrap Pearson skipped correlation and bootstrap Spearman skipped correlation). Significant correlations were further tested for survival of multiple comparison correction by controlling for family-wise error rate using maximum statistics through permutation testing (Groppe et al., 2011).

### Linear discriminant analysis (LDA)

The performance of LDA was evaluated using 10-fold cross validation (a model validation technique for assessing how the results of a model will generalize to an independent data set). To perform this cross validation, both TD and ASD Subjects (35 total) were randomly partitioned into 10 equal size subsamples. Of the 10 subsamples, 9 subsamples were used as training data for model learning and then applied on the remaining subsample to test the validity of the model. The cross-validation process was then repeated 10 times, with each of the subsamples used once as the validation data. Scikit-learn Machine Learning in Python (Pedregosa et al., 2011) was used for the above analysis.

### Akaike information criterion (AIC)

Given a set of models for the data, the Akaike Information Criterion (AIC) is a measure that assesses the quality of each model, relative to the remaining models in the set. The chosen model minimizes the Kullback-Leibler distance between the model and the ground truth. AIC takes into account both descriptive accuracy and parsimony, since it carries a penalty for increasing the number of free parameters. The model with the lowest AIC is considered the best model among all models specified for the data at hand. The absolute AIC values are not particularly meaningful since they are specific to the data set being modeled. The relative AIC value (ΔAIC_i_ = AIC_i_ – min{AIC_p_}) is used to rank models: ΔAIC_i_ < 2 suggest that models are basically equivalent, whereas a ΔAIC_i_ > 10 indicates that the model with the minimum AIC (min{AIC_p_}) is significantly better than the alternative model (Akaike, 1992).

### Statistical analyses on cortical surface

Our statistical analyses (Figure 3) were based on cluster-based statistics which is a non-parametric method (Maris and Oostenveld, 2007; Maris et al., 2007) that also corrects for multiple comparisons. We used 1000 permutations and the test statistics used were Wilcoxon Rank Sum test.

## Author contributions

SK and TK designed research; SK, FM, HB, SG, KG collected the data; SK, JH, FM, KM, MK, and MH analyzed the data; and SK, JH, MH, and TK wrote the paper. All authors reviewed the manuscript.

## Funding

This work was supported by grants from the Nancy Lurie Marks Family Foundation (TK, SK, MK), Autism Speaks (TK), The Simons Foundation (SFARI 239395, TK), The National Institute of Child Health and Development (R01HD073254, TK), The National Center for Research Resources (P41EB015896, MH), National Institute for Biomedical Imaging and Bioengineering (5R01EB009048, MH), and the Cognitive Rhythms Collaborative: A Discovery Network (NFS 1042134, MH).

### Conflict of interest statement

The authors declare that the research was conducted in the absence of any commercial or financial relationships that could be construed as a potential conflict of interest.

## Supplementary material

Figure S1 **ROCs showing performance of statistical classifier**. We evaluated the performance of the classifier using the standard approach of measuring the area under the curve (AUC), where an AUC of 0.5 represents chance (dashed blacked line). Orange line, represent average ROC curve for 10-fold validation, standard error of the folds is represented as shaded area around the line. (AUC = 0.95).

Movie M1 **Rotating visualization of the 4D depiction of the LDA shown in Figure 5**.

Table T1 **Participants in experimental paradigm**. As expected, only ADOS scores and Touch scores were significantly different between the groups.
