# Supplementary figures and images for: Altered Onset Response Dynamics in Somatosensory Processing in Autism Spectrum Disorder
Source: Front Neurosci. 2016 Jun 8;10:255. doi: 10.3389/fnins.2016.00255 (PMC4896941; doi:10.3389/fnins.2016.00255)

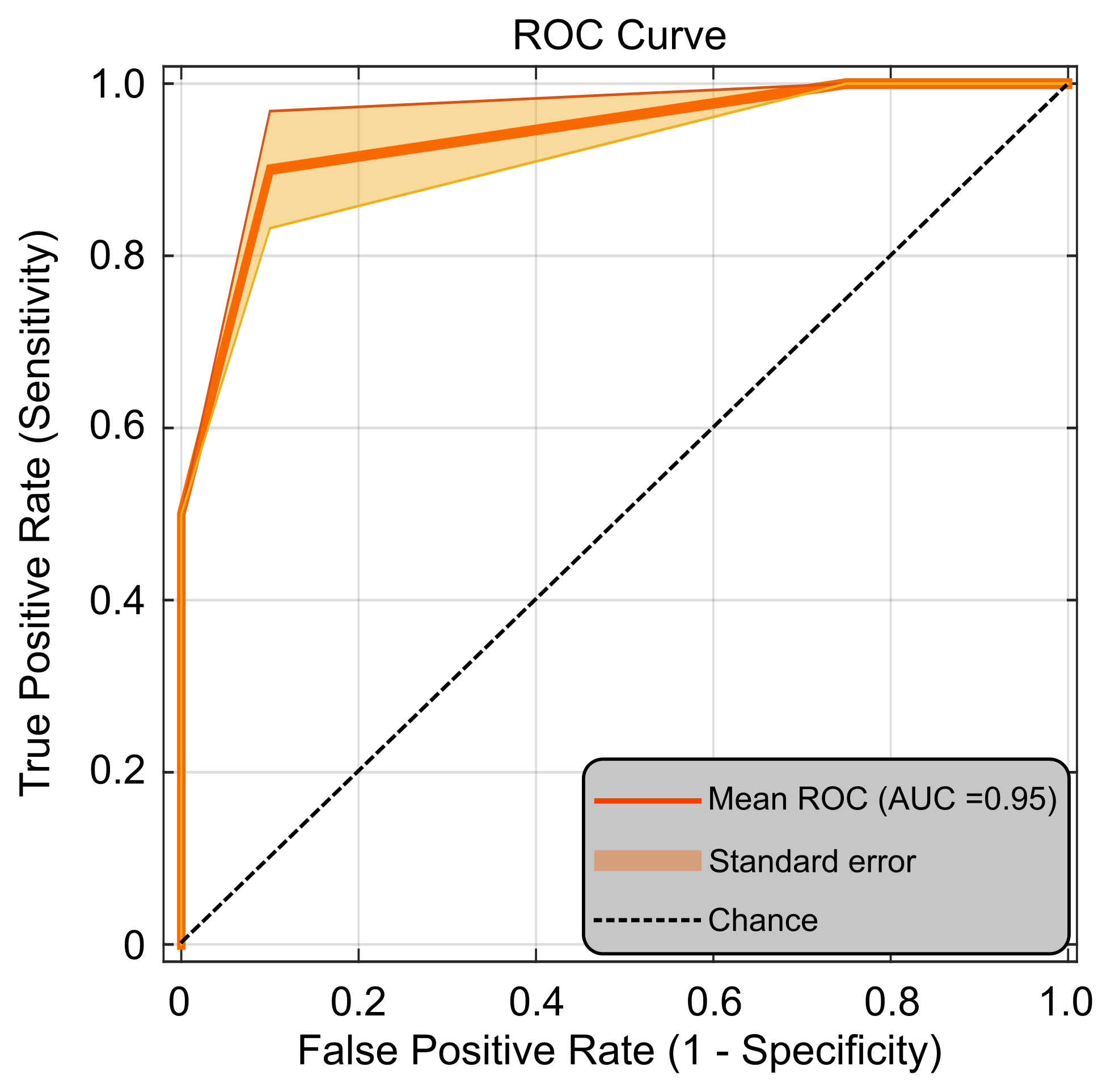

Supplement: Figure S1 — ROCs showing performance of statistical classifier. We evaluated the performance of the classifier using the standard approach of measuring the area under the curve (AUC), where an AUC of 0.5 represents chance (dashed blacked line). Orange line, represent average ROC curve for 10-fold validation, standard error of the folds is represented as shaded area around the line. (AUC = 0.95). [file Image1.PNG]
