# Supplementary material for: Altered Onset Response Dynamics in Somatosensory Processing in Autism Spectrum Disorder
Source: Front Neurosci. 2016 Jun 8;10:255. doi: 10.3389/fnins.2016.00255 (PMC4896941; doi:10.3389/fnins.2016.00255)
Supplement: Table T1 — Participants in experimental paradigm. As expected, only ADOS scores and Touch scores were significantly different between the groups. [file Table1.docx]

|  | **ASD (*n=15*) Mean (SD), Range** | **TD (*n=20*) Mean (SD), Range** | **p-value** |
| --- | --- | --- | --- |
| **Age** | 11.4 (3.7), 8-18 | 11.9 (2.8), 8-18 | 0.6 |
| **ADOS Combined** | 11.5 (3.5), 7-19 | 2.2 (1.8), 0-5 | 0.000000002 |
| **ADOS Social** | 7.8 (2.5), 5-13 | 1.5 (1.3), 0-4 | 0.00000002 |
| **ADOS Communication** | 3.7 (1.2), 2-6 | .7 (.7), 0-2 | 0.00000003 |
| **Verbal IQ** | 110 (15.8), 83-141 | 110.6 (15.6), 80-141 | 0.9 |
| **Nonverbal IQ** | 101.5 (19.2), 73-144 | 110.4 (17.0), 77-140 | 0.2 |
| **Touch Score** | 55.7 (12.5), 34-74 | 81.4 (8.1), 60-90 | 0.0000005 |

**Table T1: Participants in experimental paradigm.** As expected, only ADOS scores and Touch scores were significantly different between the groups.
